# Study of Regulatory T-Cells in Patients with Gastric Malt Lymphoma: Influence on Treatment Response and Outcome

**DOI:** 10.1371/journal.pone.0051681

**Published:** 2012-12-19

**Authors:** Mar García, Beatriz Bellosillo, Blanca Sánchez-González, Francesc García-Payarols, Agustin Seoane, Ana Maria Ferrer, Eva Gimeno, Luis Eugenio Barranco, Ariadna Torner, Francesc Solé, Carles Besses, Sergi Serrano, Antonio Salar

**Affiliations:** 1 Departments of Pathology, Hospital del Mar, Barcelona, Spain; 2 Department of Hematology, Hospital del Mar, Barcelona, Spain; 3 Department of Gastroenterology, Hospital del Mar, Barcelona, Spain; 4 IMIM (Hospital del Mar Medical Research Institute), Barcelona, Spain; University of Barcelona, Spain

## Abstract

**Purpose:**

FOXP3+ regulatory T cells (Treg) play an essential role in modulating host responses to tumors and infections. The role of these cells in the pathogenesis of MALT lymphomas remains unknown. The aims of the study were to quantify the number of infiltrating FOXP3+ and CD3+ cells in patients with gastric MALT lymphoma at diagnosis and to study kinetics of these cells and CD20+ tumor cells after treatment and during long-term follow-up.

**Methods:**

FOXP3+, CD3+ and CD20+ cells were analyzed by immunohistochemistry and the number of cells was quantified using a micrometric ocular. Samples of 35 patients with gastric MALT lymphoma at diagnosis and after treatment were included. Diagnostic samples were compared to 19 cases of chronic gastritis and diffuse large B-cell lymphoma (DLBCL) of the stomach.

**Results:**

The median number of FOXP3+ infiltrating cells was higher (27 cells/cm^2^) in gastric MALT patients than in DLBCL (10 cells; p = 0.162) but similar to chronic gastritis (20 cells; p = 0.605). No characteristic or specific distribution pattern of infiltrating FOXP3+ cells was found. Gastric MALT lymphoma patients responding to bacterial eradication therapy had higher number of FOXP3+ cells at study entry. Kinetics of both infiltrating FOXP3+ cells and tumor CD20+ cells were strongly dependent on the treatment administered.

**Discussion:**

Gastric MALT lymphomas have a number of Treg cells more similar to chronic gastritis than to DLBCL. Patients with higher number of tumor infiltrating FOXP3+ cells at study entry seem to have better response to antibiotics. Kinetics of Treg and tumor cells are influenced by type of treatment.

## Introduction

Gastric mucosa-associated lymphoid tissue (MALT) lymphoma is an indolent B-cell neoplasm associated with *H. pylori* infection in over 70–80% of cases. It has been demonstrated that continued proliferation of gastric MALT lymphoma cells depends on the presence of T-cells specifically activated by *H. pylori* antigens [1]. The induction of remissions with antibiotics to eradicate *H. pylori* illustrates how important this pathogenic mechanism is. However, the acquisition of some translocations confers resistance to antibiotics in some patients and, as a result, these cases are usually treated with alkylating agents, purine analogs, and recently, rituximab.

Multiple T-cells have been shown to be important for immune homeostasis, being regulatory T cells (Treg) a subset of critical importance. Currently, the most widely used and reliable Treg marker is the forkhead box transcription factor Foxp3, which is expressed specifically in CD4+ Tregs and which is essential for their lineage identity and suppressive function [2]–[4]. In patients with cancer, Treg cells have been reported to have influence on outcome and this may be due, at least in part, through the ability of these cells to suppress antitumor immune response either through TCR activation, cell-to-cell contact, or by producing TGF-beta [5]. Moreover, intratumoral FOXP3+ T cells have been shown to migrate or be induced in response to chemokines produced by malignant B cells in some lymphomas [6], [7]. Furthermore, Tregs can also induce activated macrophages, the so-called M2 type which are associated with protumoral immunity [8]. Recent evidence suggests that not only is the quantity of tumor-infiltrating Treg cells important for outcome in some types of lymphoma, but also its immunoarchitectural distribution [9]–[18].

Another important feature is the fact that the impact of the microenvironment in the prognosis may depend on the type of treatment administered [19]. Then, since Treg cells play an essential role in modulating host responses to tumors and infections, it could be hypothesized that Treg cells might be important in the cross-talk between neoplastic MALT B-cells and T-cells specifically activated by *H. pylori* antigens. According to this theory, it has been shown recently in a murine model of *H. pylori*-induced gastric MALT lymphoma an overrepresentation of Treg cells with highly suppressive activity compared with conventional CD4+ T cells in the tumor microenvironment and their active recruitment through tumor B-cell derived chemokines CCL17 and CCL22 [20]. In vivo, the role of Tregs cells in gastric MALT lymphoma has not been studied yet.

To further characterize the role of Treg cells in gastric MALT lymphoma, we analyzed a consecutive series of patients with gastric MALT lymphoma by quantifying the number of infiltrating CD3+ and FOXP3+ cells at diagnosis, and by describing the distribution pattern of FOXP3+ cells within the tumor. In order to know whether the microenvironment in gastric MALT lymphoma was more like a reactive condition or an aggressive lymphoma, we carried out the same study on samples with chronic gastritis and diffuse large B-cell lymphoma (DLBCL) of the stomach. In addition, to gain insight into the possible influence of therapy on microenvironment and tumor cells in gastric MALT lymphoma, we evaluated the kinetics of CD3+, FOXP3+ and CD20+ cells by immunohistochemistry after treatment and during long-term follow-up.

## Materials and Methods

### Inclusion criteria and work-up

Patients consecutively diagnosed with gastric MALT lymphoma in a single institution between 2000 and 2010 were included in the present study, with the only criterion for inclusion being the availability of histologic material. All of the cases were reviewed and classified according to the criteria of the World Health Organization (WHO) Classification [21]. *H. pylori* status was determined by histology and/or breath test, or serology in negative cases. Tissue and clinical data were retrieved according to the regulations of the institutional review board (Comitè Ètic d'Investigació Clínica del Parc de Salut Mar CEIC-PSMAR) and data protection laws. All patients underwent disease extension study that included medical history, physical examination, laboratory, computed tomography scans of chest, abdomen and pelvis; unilateral bone marrow biopsy and upper endoscopy with multiple gastric biopsies. In addition, patients diagnosed after 2005 underwent upper ultrasound endoscopy. Stage was determined according to Lugano system [22]. The first response evaluation was done 1–2 months after finishing treatment, and every 6 months during the first two years and annually thereafter. Response was classified according to the definitions recommended by the International Workshop to Standardize Response Criteria for non-Hodgkin's lymphomas [23]. Criteria of Wotherspoon et al was used to establish the diagnosis of gastritis and MALT lymphoma as well as to evaluate histologic response [24].

### Patient characteristics

The median age of the 35 patients that met the criteria for inclusion in the study was 64 years (range, 32 to 83 years), and 54% of patients were male. The baseline characteristics are summarized in Table 1. It is noteworthy that 32 patients (91%) had localized gastric involvement, 23 with stage I and 9 with stage II according to Lugano system [22]. *H. pylori* infection was present in 17 cases (49%) at the time of study entry. Ten out of 34 patients (29%) had t(11;18)(q21;q21). BCL10 nuclear expression was found in 12 cases (34%), although two of these cases were not associated to t(11;18)(q21;q21) nor to t(1;14). Twelve patients had been treated with eradication antibiotic therapy against *H. pylori* as their sole initial treatment in other centers before study entry. Triple therapy with omeprazole, amoxicillin and clarithromycin was the most common. Chemotherapy was administered according to standard practice or clinical trials (table 2).

**Table 1:** Clinical characteristics of gastric MALT lymphoma patients at study entry.

Number of patients	35
Median age (range)	64 (32–83)
Gender	
Male	19 (54%)
Female	16 (46%)
Lugano Stage	
I	23 (66%)
II	9 (26%)
IV	3 (9%)
B-symptoms	2 (6%)
*H. pylori*	17 (49%)
t(11;18)(q32;q32)	10 (29%)
BCL-10 nuclear expression	12 (34%)

**Table 2:** Number and types of treatments followed by the patients included in the study.

	N
**Number of treatment lines by patient**	
1	32
2	5
3	1
ND	3
**Type of treatment**	
Eradication therapy anti-HP	12
Single or combined chemotherapy without rituximab	5
Rituximab alone or CHOP-like with rituximab	4
Fludarabine	8
Fludarabine or bendamustine with Rituximab	9
Interferon/ribavirin	1

ND, not done.

### Immunohistochemistry and FOXP3 quantification

Tissue samples were fixed in formalin and embedded in paraffin by routine methods. A series of immunohistochemical stains were performed in consecutive sections including CD20 (L26)(DAKO), CD3 (F7.2.38)(Novocastra), FOXP3 (236A/E7)(eBioscience), bcl-10 (clone 151.1, MBL Medical and Biological Laboratories, Nagoya, Japan) and Ki67(MIB1)(DAKO). Immunostaining was performed using a Dako Ptlink autostainer and the EnVision Flex polymer detection system (Dako, Glostrup, Denmarck) using diaminobenzidine as a chromogen in this reaction.

The number of FOXP3-positive cells was quantified in whole-tissue sections of all samples. The total number of FOXP3+ infiltrating cells was quantified using a micrometric ocular (WPK 10× mn) that has a 10 mm linear scale divided to 100 parts and cell counts were referred to 1 mm^2^. The number of CD20+ tumor cells and CD3+ cells was also quantified with the same methodology and using the same areas that were evaluated for FOXP3.

### Cytogenetic and clonality studies

The t(11;18)(q21;q21) was analyzed on all diagnostic specimens using fluorescent in situ hybridization (FISH) with the commercially available break-apart LSI-MALT1 probe (Vysis, Downers Grove, IL) or by reverse transcriptase-PCR (RT-PCR) for API2/MALT1 fusion product, as previously described [25]. In postreatment specimens, only initially positive cases were reassessed by RT-PCR. In those cases with nuclear staining for bcl-10 but without t(11;18)(q21;q21), the t(1;14)(p22;q32) was analyzed using FISH with the commercially available break-apart BCL10 DNA probe (Dako, Glostrup, Denmarck).

Immunoglobulin gene rearrangements were analyzed as previously described [25]. In brief, deoxyribonucleic acid was obtained from paraffin-embedded tissue blocks using the QIAamp Tissue Kit (QIAGEN GmbH, Hilden, Germany). For heavy immunoglobulin chain (IgH) gene analysis, DNA was amplificated by PCR using consensus primers specific for framework (FR)1, FR2, FR3 following in both analysis the BIOMED-2 protocol. One of the primers was fluorescent dye-labeled and analysis of the amplified products was performed by capillary electrophoresis in an automated DNA sequencer (ABIPrism 3100, Applied Biosystem, Foster City, CA). Duplicate PCR amplifications were performed.

### Statistical Analysis

The main characteristics at study entry and during follow-up, including immunohistochemistry parameters, were recorded and analyzed for prognostic significance. Statistical comparisons were made using the Mann–Whitney U test or Fisher's exact test for nonparametric values, or the chi-square test, as appropriate. P values of less than 0.05 for two-sided tests were considered statistically significant. Curves of FOXP3, CD3 and CD20 evolution were constructed using base package of R. The statistical software packages SPSS (version 15.0) and R (R Development Core Team, R Foundation for Statistical Computing, Vienna, Austria, 2008) were used.

## Results

### Distribution pattern of B and T cells in chronic gastritis, gastric MALT lymphoma and DLBCL of the stomach

In addition to gastric MALT lymphoma cases, we analyzed 12 patients with chronic gastritis according to Wotherspoon criteria [24] (7 associated to *H. pylori* infection and 5 without evidence of *H. pylori* infection) and 7 patients with gastric diffuse large B-cell lymphomas transformed from MALT, in order to set the immunohistochemic findings in different lymphoid conditions of the stomach.

At diagnosis, the median (range) number of CD20+ cells in patients with gastric MALT lymphoma was 700 (85–1000) cells/mm^2^.These figures were different to those observed in patients with DLBCL transformed from MALT (484 (207–760) cells/mm^2^; p = 0.041) or chronic gastritis (304 (16–550) cells/mm^2^; p<0.0001). The number of infiltrating CD3+ cells was 125 (30–258) cells/mm^2^ in MALT, 66 (9–226) cells/mm^2^ in DLBCL and 176 (73–499) cells/mm^2^ in chronic gastritis (table 3). FOXP3+ cells could be assessed in all gastric MALT lymphoma cases. We were not able to find any characteristic or specific distribution pattern of FOXP3+ infiltrating cells. When germinal centers were present, FOXP3+ cells were more numerous within them, whereas no specific pattern of infiltration could be observed when the infiltrate was uniform. The median (range) number of FOXP3+ infiltrating cells was higher (27 (1–110) cells/mm^2^) in gastric MALT patients than in DLBCL (10 (1–22); p = 0.162) but similar to chronic gastritis (20 (1–133); p = 0.605). The proportion of FOXP3+ as a fraction of all CD3+ cells was not different between MALT lymphoma and DLBCL or chronic gastritis (p = 0.826 and p = 0.222, respectively).

**Table 3:** Immunohistochemistry by gastric condition (cells/mm^2^).

	MALT lymphoma HP+ (N = 17)	MALT lymphoma HP− (N = 18)	DLBCL (N = 7)	CG HP+ (N = 7)	CG HP− (N = 5)
CD20+	700(85–1000)	750(174–1000)	484 (207–760)	430 (16–550)	191 (122–534)
FOXP3+	28 (1–110)	27 (1–100)	10 (1–22)	30 (11–97)	15 (1–133)
CD3+	151 (44–243)	109.5 (30–258)	66 (9–226)	243 (73–376)	140 (101–499)
Ratio FOXP3/CD3	22.3%(0.8–63%)	19.9%(0.51–53.76%)	14% (4–40%)	12% (6–27%)	11% (1–27%)

MALT: mucosa-associated lymphoid tissue; DLBCL: diffuse large B-cell lymphoma; CG: chronic gastritis; HP: *Helicobacter pylori*.

The median number of CD20+ tumor cells and FOXP3+ infiltrating cells was similar among gastric MALT lymphomas with or without t(11;18). In this group, HP status did not have influence in the number of CD20+, CD3+ and FOXP3+ infiltrating cells (p = 0.551; p = 0.552; p = 0.564). We did not observe differences in the number of FOXP3+, CD3+ or ratio FOXP3+/CD3+ according to clinical or biologic parameters in MALT (data not shown) with the exception that the median number of CD3+ cells was higher in patients with stage I (175 (44–258) cells/mm^2^) than with stage II–IV (103 (30–186) cells/mm^2^)(p = 0.021).

### Response to therapy

Thirty-nine treatments were analyzed: 32 patients received 1 treatment, 5 patients received 2 and 1 patient 3. Type of treatment was not available in three patients.

Treatment regimens are shown in Table 2. In summary, 13 cases were treated with anti-infectious therapy alone, 9 with single or combined agent chemotherapy with or without rituximab and 17 with fludarabine or bendamustine with or without rituximab.

Overall response rate was 85% (77% complete remission (CR)). Age older than 60 years was the only factor associated with a worse response to therapy (p = 0.049). Patients carrying t(11;18) did not response worse since all of them did receive immunochemotherapy with or without antibiotics according to their *H. pylori* status. Only one patient with strong nuclear expression of bcl10 without t(11;18) or t(1;14) did not respond to 3 lines of immunochemotherapy. In the whole group, the number of cells CD20+, CD3+, FOXP3+ and FOXP3/CD3 ratio was not associated with different response to treatment. However, interestingly, the number of FOXP3+ cells did influence response in those patients treated with anti-infectious therapy alone. The median number of FOXP3+ cells in responding patients was higher than in non responders (40 (3–110) cells/mm^2^) vs 3 (1–5) cells/mm^2^; p = 0.048). In addition, the ratio of FOXP3/CD3 was higher in responders (29%(4–64%) vs 2%(1–3%); p = 0.03).

Histologic and molecular overall remissions at the end of therapy were 72% and 56%, respectively.

### Kinetics of CD20+ tumor cells and infiltrating FOXP3+ and CD3+ cells

**Figure 1:**
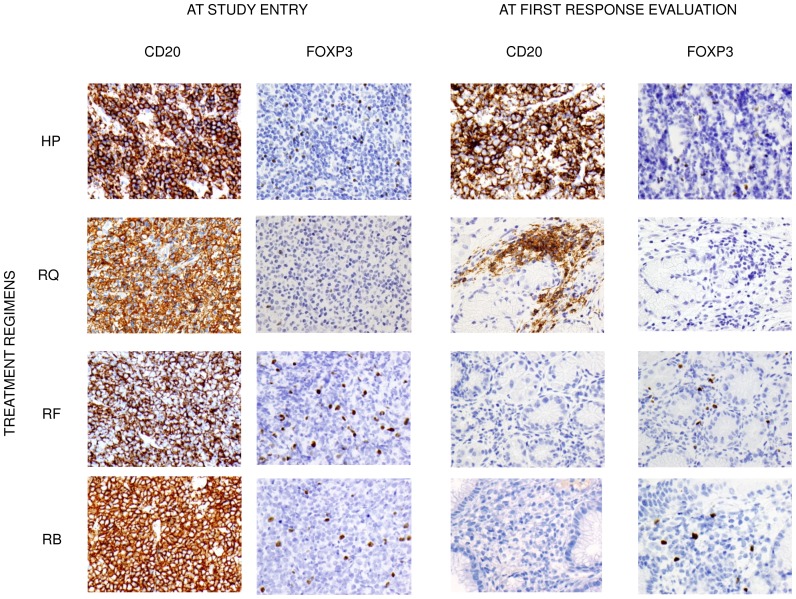
Immunohistochemistry for CD20 and FOXP3 at study entry and at first response evaluation (1–2 months after finishing treatment). Gastric biopsies samples from 4 gastric MALT lymphoma patients treated with different schedules. HP: erradication therapy alone; RQ: Rituximab+CHOP; RF: Rituximab+Fludarabine; RB: Rituximab+Bendamustine.

**Figure 2:**
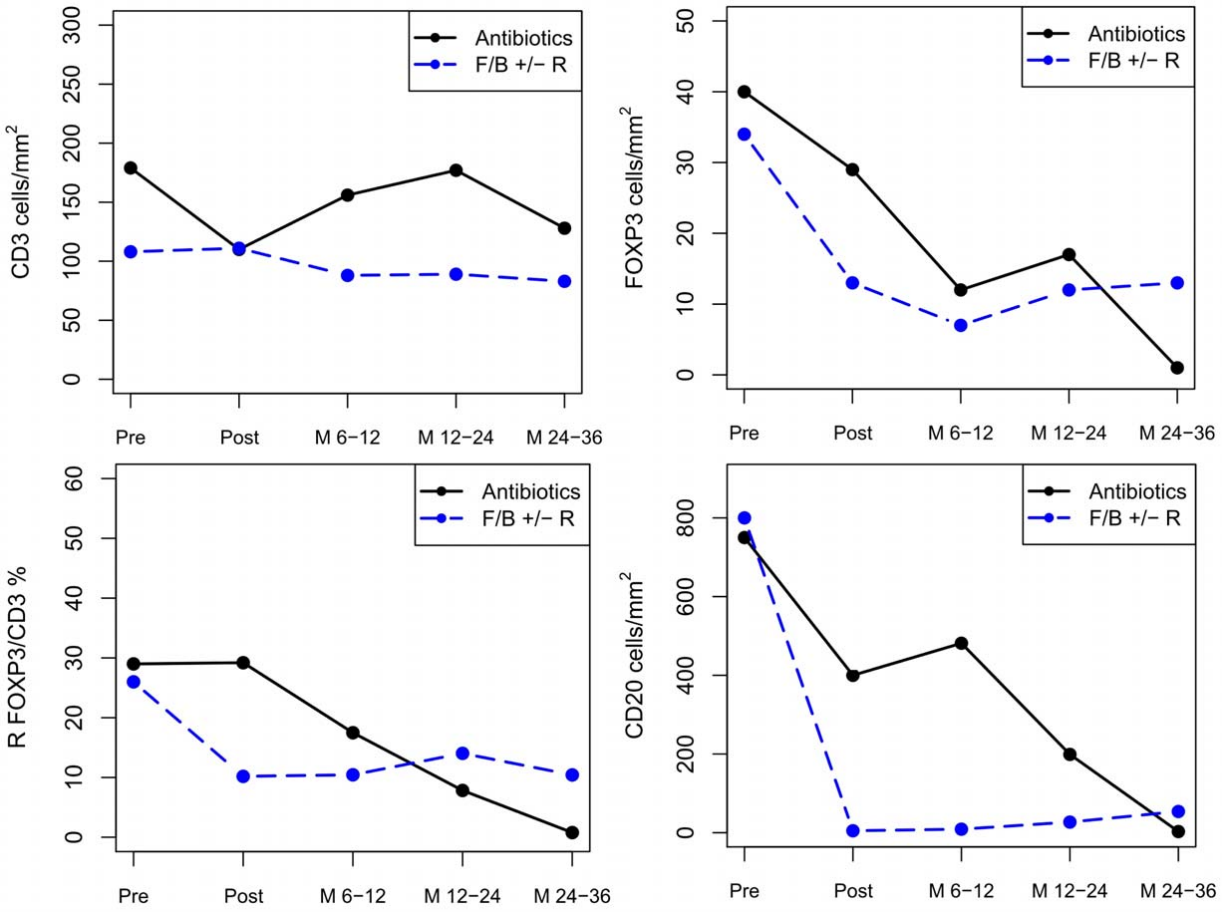
Kinetics of CD3+, FOXP3+, ratio FOXP3+/CD3+ and CD20+ cells by immunohistochemistry in responding patients. Median CD20+ cells were significantly different between cases treated with antibioticis and those treated with fludarabine or bendamustine with or without Rituximab at first response evaluation (p = 0.001) but not later during follow-up.

Overall, the median number of CD20+ cells was significantly reduced after treatment but, as expected, CD20+ depletion was deeper in responding patients (p<0.0001). Treatment type had a strong impact on CD20 kinetics. Fludarabine or bendamustine with or without rituximab induced a quick and profound depletion in the number of CD20+ tumor cells in comparison with the other treatments (figure 1 and 2). In responding patients treated with anti-infective therapy, CD20 depletion was progressive and at least for more than 1 year, reaching similar values than those achieved with fludarabine or bendamustine. t(11;18) or bcl10 status did not have impact in CD20 kinetics.

At the end of treatment, FOXP3+ infiltrating cells decreased from a median of 31 cells/cm^2^ to 15 cells/cm^2^ in responding patients, but no changes were seen in non responding cases. Although FOXP3+ cell depletion at the end of treatment was slightly deeper in cases treated with fludarabine or bendamustine than in those treated with antibiotics, FOXP3+cells continue to falling up to one year in both treatment groups.

No significant reduction of CD3+ cells was observed after treatment. Even in patients treated with fludarabine or bendamustine with or without Rituximab, the median number of CD3+ cells was similar pre and post therapy (108 cells/mm^2^ vs 96 cells/mm^2^, p = 0,831). Moreover, the ratio of FOXP3/CD3 did not vary after treatment in any group.

After a median follow-up of 54 months (range, 1–8 years), 4 patients have relapsed at 3, 5, 23 and 31 months. Samples at relapse were available in all 4 cases and we could not find any difference in the number of cells (CD20+, CD3+ or FOXP3+) between diagnosis and relapse (table 4). No death related mortality has been observed.

**Table 4:** Data of relapsed patients both at diagnosis and at relapse.

	Treatment	Time to relapse*	At diagnosis	At relapse
			CD20+/CD3+/FOXP3+	CD20+/CD3+/FOXP3+
Patient 1	HP	3	700/114/28	1110/30/29
Patient 2	F	5	800/186/100	628/250/210
Patient 3	HP	23	750/184/67	700/130/37
Patient 4	Chemo	31	660/125/1	855/70/10

HP: erradication therapy; F: Fludarabine; Chemo: Chemotherapy.

* Time to relapse in months.

## Discussion

Tumor cell microenvironment is now thought to play a relevant role in the biology of B-cell lymphomas. It has been shown that lymphoma-infiltrating FOXP3+ cells vary between different lymphoma types and these cells may represent important lymphoma/host microenvironment-modulators [26], [27]. Our study provides further information on tumor-infiltrating FOXP3+ cells in lymphomas and other gastric conditions, in particular in gastric MALT lymphoma with special emphasis in response after therapy and in long term follow-up.

We first evaluated tumor-infiltrating FOXP3+ cells in gastric MALT lymphomas and, to study them in the context of other lymphoid conditions of the stomach, we analyzed samples with chronic gastritis, as precursor lesions preceding gastric MALT lymphomas, and gastric diffuse large B-cell lymphoma, as late event lesions resulting from progression of gastric MALT lymphoma. Interestingly, the number of FOXP3+ infiltrating cells in gastric MALT lymphoma was more like chronic gastritis than transformed lymphomas. Our findings suggest the hypothesis that immune homeostasis in gastric MALT lymphoma is more similar to chronic gastritis but different to the regulatory mechanisms which take over the events leading to progression. In fact, the lower number of FOXP3+cells in gastric DLBCL evolving from MALT observed by us was 2.7 fold, a figure that is similar to the decreased fold observed in FL and transformed FL in two previous studies [14], [15].

We next investigated the distribution pattern of FOXP3+ cells in lymphoma and chronic gastritis samples. In both settings, T reg cells were more numerous within germinal centers, when they were present, but we could not find a specific distribution of Treg infiltration when the lymphoid infiltrate was diffuse. Therefore, in contrast to previous observations in follicular lymphoma, no characteristic architectural pattern of FOXP3+ cells was found in gastric MALT lymphoma [9].

We further investigated the value of FOXP3+ infiltrating cells at diagnosis to predict treatment response. We analyzed our data splitting them up into several treatment groups because previous studies in FL have shown that specific therapeutic regimens decisively influenced the prognostic impact of the microenvironment [19]. Our results showed that gastric MALT lymphoma patients responding to eradication therapy had higher number of FOXP3+ cells. However, in our patients treated with chemotherapy or immunochemotherapy, we were not able to detect influence in the response rate according to the number of FOXP3+ cells at diagnosis. We also observed in our study that the total number of CD3+ cells did not have impact in the response in any therapeutic group, similar to that seen in FL. Our results suggest that Treg cells have stronger impact in treatments that are not directly targeted against the tumor cells. This is probably due to the role of Tregs in modulating tumor growth through different mechanisms. It is well-known that these cells are able to suppress the activity of antigen-presenting T-cells. Our findings showing that the number of Treg cells has a positive influence in the response to antibiotic therapy raise the possibility of a suppressive influence of these Tregs on the T-cell population responsible of tumor growth maintenance through the presentation of *H. pylori* antigens.

We also analyzed the impact of treatment on the kinetics of FOXP3+, CD3+ and CD20+ cells determined by immunohistochemistry in gastric tissue. Our MALT lymphoma patients were treated according to current standard practice but some of our cases were treated with drugs such as fludarabine or bendamustine, with or without rituximab, in the context of clinical trials [28]–[29]. We observed a deep depletion of FOXP3+ cells at gastric tissue in patients responding to eradication therapy and to fludarabine or bendamustine, with or without rituximab, achieving its lowest values at months 6–12 and maintaining at the same magnitude during follow-up. Interestingly, depletion of T reg cells was slightly slower in patients treated with antibiotics than in those cases treated with purine analog structure drugs. In a recent published study in a similar population, an increase in density of FOXP3+ cells after treatment with oral fludarabine until a CR was documented [30]. With regard to CD3+ cells, we observed a modest reduction of these cells at gastric tissue after treatment with eradication therapy in responding cases. However, unexpectedly, the number of CD3+ cells at the gastric tissue was not reduced at after treatment with fludarabine or bendamustine, even though these patients had intense lymphocytopenia in the peripheral blood (data not shown). Similar results were observed by de Boer et al [30]. Therefore, these observations highlights the fact that different type treatments influence T cells at peripheral blood and at gastric tissue in a very different way.

Through the quantification of CD20+ tumor cells, we have observed a remarkable different pattern in the kinetics of these cells depending on whether the administered treatment was antibiotics or chemotherapy. Both treatments achieved a profound elimination of tumor cells, but whereas in patients treated with chemotherapy this is achieved immediately after treatment, in patients treated with antibiotics occurred much more slowly over several months. Our findings show that some remissions can occur several months after finishing therapy and are in line with the clinical recommendation to wait several months in patients treated with antibiotics before starting a new therapy, unless a clinical relapse occurs [31].

In summary, our results show that gastric MALT lymphoma patients with high number of tumor infiltrating FOXP3+ cells have better response to eradication therapy. Kinetics of both infiltrating FOXP3+ cells and tumor CD20+ cells were strongly dependent on the treatment administered, showing different patterns between cases treated with antibiotics and those treated with immunochemotherapy.
